# Population mixing and leukaemia in young people around the La Hague nuclear waste reprocessing plant

**DOI:** 10.1038/sj.bjc.6600529

**Published:** 2002-09-23

**Authors:** O Boutou, A-V Guizard, R Slama, D Pottier, A Spira

**Affiliations:** Ecole Nationale de la Santé Publique, département Egeries, avenue du Professeur Léon Bernard, CS 74312, 35043 Rennes cedex, France; INSERM Research Unit 569, 82 rue du Général Leclerc, 94276 Le Kremlin Bicêtre, France; Registre des cancers de La Manche (ARKM), hôpital Louis Pasteur, 46 rue du Val de Saire, 50102 Cherbourg cedex, France; GRECAN, UPRES EA 1772, Université de Caen, esplanade de la paix, 14032 Caen, France

**Keywords:** childhood leukaemia, rural population mixing, geographical study, Poisson regression, La Hague

## Abstract

In order to investigate for an association between population mixing and the occurrence of leukaemia in young people (less than 25 years), a geographical study was conducted, for the years 1979 to 1998, in Nord Cotentin (France). This area experienced between the years 1978 and 1992 a major influx of workers for the construction of a nuclear power station and a new nuclear waste reprocessing unit. A population mixing index was defined on the basis of the number of workers born outside the French department of ‘La Manche’ and living in each ‘commune’, the basic geographical unit under study. The analyses were done with indirect standardisation and Poisson regression model allowing or not for extra-Poisson variation. Urban ‘communes’ were considered as the reference population. The Incidence Rate Ratio was 2.7 in rural ‘communes’ belonging to the highest tertile of population mixing (95% Bayesian credible interval, 95%BCI=1.2–5.9). A positive trend was observed among rural strata with increasing population mixing index (IRR for trend=1.4, 95%BCI=1.1–1.8). The risk became stronger for Acute Lymphoblastic Leukaemia in children 1–6 years old in the highest tertile of population mixing (IRR=5.5, 95%BCI=1.4–23.3). These findings provide further support for a possible infective basis of childhood leukaemia.

*British Journal of Cancer* (2002) **87**, 740–745. doi:10.1038/sj.bjc.6600529 www.bjcancer.com

© 2002 Cancer Research UK

## 

The increased risks of leukaemia in young people living in the vicinity of the nuclear waste reprocessing plants at Sellafield, Dounreay and La Hague have been the subject of extensive scientific study (Black, 1984; Gardner *et al*, 1990; Guizard *et al*, 2001; Heasman *et al*, 1986; Pobel and Viel, 1997; Urquhart *et al*, 1991; Viel *et al*, 1995). Radiological assessments concluded that the levels of radiation in these areas were far below those necessary to account for the observed excesses (COMARE, 1988, 1996; Rommens *et al*, 2000). An association between cases of leukaemia and pre-conceptional irradiation of their fathers while working at Sellafield was hypothesised (Gardner *et al*, 1990), but this could not account for the excess around Dounreay (Urquhart *et al*, 1991) and La Hague (Pobel and Viel, 1997).

The areas near Sellafield and Dounreay are remarkable because of their geographic isolation and large population influx (Kinlen, 1988). Kinlen postulated that in such areas the herd immunity to an unknown and widespread infectious agent could be lower than average and that a large population influx might have been conducive to epidemics. Childhood leukaemia could be a rare response to this common infection (Kinlen, 1988). This hypothesis, known as the population mixing hypothesis, is supported by a series of studies, the four marked with asterisks demonstrating transmission by adults (Kinlen and Hudson, 1991^*^, Kinlen *et al*, 1990, 1991, 1993^*^, 1995^*^; Kinlen, 1995; Dickinson and Parker, 1999; Kinlen and Balkwill, 2001^*^).

The Nord Cotentin region shares some characteristics with the Sellafield and Dounreay regions: it used to be an isolated and rural area which experienced a big influx of population between the years 1978 and 1992 with the construction of the Flamanville nuclear power station and a new nuclear waste reprocessing unit on the La Hague site. The objective of the study was to investigate for an association between population mixing and leukaemia among young people living in the Nord Cotentin region.

## MATERIALS AND METHODS

The Nord Cotentin region (French department of ‘La Manche’), situated less than 35 km from the La Hague nuclear waste reprocessing plant, is divided into 107 villages or towns called ‘communes’. This is the smallest administrative geographical unit available in France and it was used in the analysis. As the influx of workers started in the second part of 1978 with the construction of the Flamanville nuclear power plant, we chose 1979 as the beginning of the study on the assumption of a minimal lag of 6 months between exposure and induction of the leukaemia cases. The end of the study period was 1998.

The collection of leukaemia cases and the estimation of Standardised Incidence Ratios (SIR) have been described elsewhere (Guizard *et al*, 2001). Briefly, registration and validation of leukaemia cases occurring in the Nord Cotentin region were done by the La Manche cancer register (ARKM). The number of person-years at risk was calculated for each ‘commune’ by demographic interpolation between two censuses, taking into account population ageing, birth counts and death counts (Pottier, 1992). External reference incidence rates for leukaemia were calculated from published general population French cancer registers (Bas-Rhin, Doubs and Isère 1978–1992, Calvados 1978–1987, Haut-Rhin 1988–1992) (IARC, 1987; IARC, 1992; IARC, 1997). The paediatric registers of Lorraine and of the Provence-Alpes-Côtes-d'Azur and Corsica regions were also used as references for children under 15 years and ALL (Lacour, personal communication; Bernard *et al*, 1993).

Population mixing has been associated more strongly with ALL and especially, with ALL occurring during the childhood peak regularly observed in developed countries (Dickinson and Parker, 1999). Moreover, leukaemia under the age of one year differs from childhood leukaemia from a molecular point of view, possibly reflecting a different aetiology (Greaves, 1996). Prior to analysis, it was thus decided to consider different outcomes: all leukaemia in 0–24 year olds because it corresponds to the excess originally described in the Beaumont-Hague area, ALL in 1–14 year olds and ALL in 1–6 year olds because they enclose the peak of ALL.

In the last 25 years, the area of Nord-Cotentin experienced two important population mixing situations, the first one for the construction of the Flamanville nuclear power plant (1978–1986) and the second one for the building of a new nuclear waste reprocessing unit on the La Hague site (1982–1992). In terms of numbers of workers employed at the construction sites, the La Hague building site was the most important one. The Flamanville and the La Hague construction sites were planned in order to share the same construction workers and the same accommodation for the workers. We were provided with the anonymous file of all workers who spent more than 3 weeks on the La Hague construction site with details on gender, age, place of birth, dates of employment and last address of the workers during the construction period. The registration of workers began at the third quarter of 1983. No individual data was obtained for the Flamanville building site. For each ‘commune’, a population mixing index was defined as the number of male construction workers born outside the French department of ‘La Manche’ who were recorded living in the ‘commune’ divided by the number of men aged 20–59 years at the 1975 census (considered as the number of potentially active men in the ‘commune’ before plant construction).

The ‘communes’ of Nord Cotentin were stratified according to their urban or rural status using criteria defined by the French National Institute of Statistics and Economic Studies (Institut National de la Statistique et des Etudes Economiques). Rural communes were then ranked in ascending order for the proportion of construction workers among potentially active men in 1975. By summing the expected number of leukaemia cases in rural areas, three categories with approximately similar expected numbers were created: the first one referred to rural ‘communes’ with low population mixing, the second one to rural ‘communes’ with intermediate population mixing and the last one to rural ‘communes’ with high population mixing. Urban ‘communes’ were chosen as the reference population.

Three different statistical methods were used to assess the association between population mixing and leukaemia at the ‘commune’ level. First, Standardised Incidence Ratios (SIR) were calculated as the ratio of the observed numbers of cases (O) to the expected numbers (E). The incidence rate ratio (IRR) was defined as the ratio of the SIR in one exposure group to the SIR in the reference group. Exact two-sided 95% confidence intervals (95% CI) for the IRR were calculated on the basis of the associated binomial probability (Breslow and Day, 1987). Tests for heterogeneity and trend in the IRR across exposure categories were computed (Breslow and Day, 1987). Second, Poisson regression analyses were performed. The number of cases observed in area i, O_i_, was assumed to follow a Poisson distribution with mean E_i_θ_i_, where θ_i_ may be thought of as the area-specific rate-ratio controlled for age and sex and E_i_ is the expected number of events. The Poisson regression model may be written as follows for a ‘commune’ i:


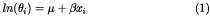


where μ is the constant, x_i_ represents the vector of explanatory variables and β is the vector of parameters. These parameters were estimated by the maximum likelihood method using STATA 6.0 (Statacorp, 1999). The goodness of fit of the Poisson regression models was assessed by a deviance statistic which could not be interpreted, however, because of the substantial proportion of ‘communes’ without any case of leukaemia (77%) (Bithell *et al*, 1995). When the disease is rare and/or the populations at risk are small, the variation in the observed number of events is greater than assumed under Poisson model. This is called extra-Poisson variation which may be due to dependence in the true disease rate between contiguous areas or to unmeasured or unknown area-level covariates.

A second model taking into account the extra-Poisson variation was computed:


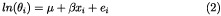


where e_i_ is a random effect allowing for extra-Poisson variation (Best *et al*, 1999; Clayton *et al*, 1993; Mollié, 1996). The random effect e_i_ was split into two components, the spatially unstructured extra-Poisson variation (heterogeneity), u_i_, and the spatially structured extra-Poisson variation (clustering), v_i_. The latter allows spatial correlation in the true disease rates (i.e. similar rates in geographically close areas). The parameters of the model were estimated in a fully Bayesian approach using Markov Chain Monte Carlo simulation. The random effects were modelled as an independent normal variable with mean zero and variance σ^2^ for u_i_ and as an intrinsic Gaussian autoregressive variable for v_i_ (convolution prior). The intrinsic Gaussian autoregression specifies the conditional prior distribution to be normal with mean depending upon the mean in the neighbouring areas and with variance inversely proportional to the number of neighbouring areas (Mollié, 1996). For each model, a burn-in period of 10 000 iterations was needed to achieve convergence as assessed by the Gelman and Rubin's diagnostic, with a further 20 000 samples providing acceptable Monte Carlo error variances. The WinBUGS 13 Bayesian software (http://www.mrc-bsu.cam.ac.uk/bugs/winbugs/contents.shtml) was used for the computation (Spiegelhalter *et al*, 1999).

## RESULTS

**Figure 1:**
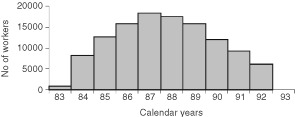
Distribution of the workers employed at least 3 weeks at the construction of the new reprocessing unit on the La Hague site (France) from 1983 to 1992: population mixing and leukaemia in young people in Nord Cotentin (France), 1979–1998

From the third quarter of 1983 to the fourth quarter of 1992, 33 437 persons worked at least 3 weeks on the La Hague building site. The construction reached a maximum in 1987 with 18 283 workers present at least 3 weeks on the site during the year (Figure 1

). The majority of them were men (92.4%) (Table 1

**Table 1:**
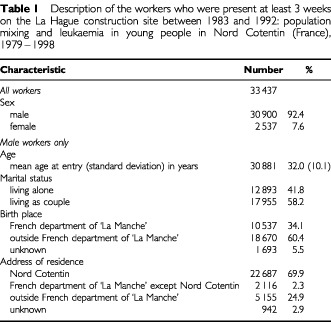
Description of the workers who were present at least 3 weeks on the La Hague construction site between 1983 and 1992: population mixing and leukaemia in young people in Nord Cotentin (France), 1979–1998

). Among male workers, the mean age at the entry on the construction site was 32 years. The rate of male workers living alone was 41.8% and 60.4% of them were born outside the French department of ‘La Manche’.

Among the 107 ‘communes’ constituting the Nord Cotentin region, six were categorised as urban, corresponding to the ‘communes’ of the Cherbourg urban community (Table 2

**Table 2:**
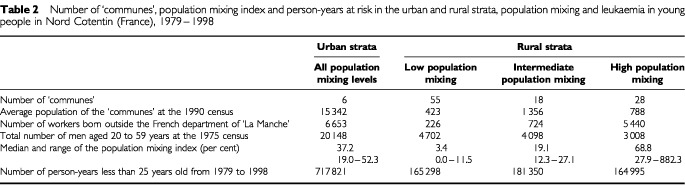
Number of ‘communes’, population mixing index and person-years at risk in the urban and rural strata, population mixing and leukaemia in young people in Nord Cotentin (France), 1979–1998

). Most of the Nord Cotentin region was classified as rural representing only 41.6% of the person-years at risk in people less than 25 years of age. The population mixing index ranged from zero in some small ‘communes’ quite far from the La Hague nuclear plant to 882.3 workers for 100 men in the Beaumont-Hague ‘commune’ situated in the immediate vicinity of the plant (Figure 2

**Figure 2:**
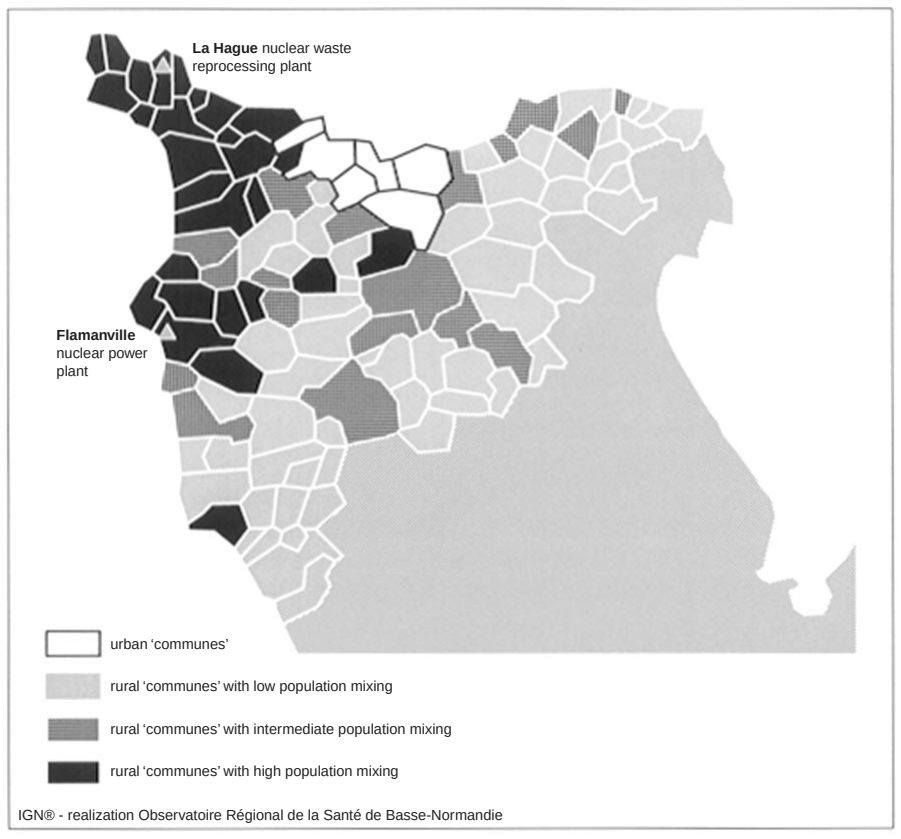
The Nord Cotentin region: urban/rural status and population mixing stratum of the ‘communes’: population mixing and leukaemia in young people in Nord Cotentin (France), 1979–1998

).

In Nord Cotentin as a whole, the observed number of leukaemia cases was consistent with the expected number (SIR=1.08; 95% CI: 0.76–1.48) (Table 3

**Table 3:**
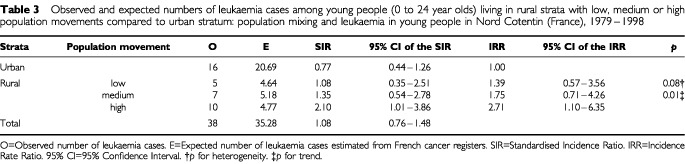
Observed and expected numbers of leukaemia cases among young people (0 to 24 year olds) living in rural strata with low, medium or high population movements compared to urban stratum: population mixing and leukaemia in young people in Nord Cotentin (France), 1979–1998

). In the rural area the most affected by population mixing, the SIR was increased (SIR=2.10; 95% CI: 1.01–3.86). The analysis by indirect standardisation showed twice as many cases of leukaemia in young people in rural ‘communes’ than in urban ones (IRR=1.95; 95% CI: 0.98–3.97). For the following results, urban ‘communes’ are considered as the reference population. The highest risk of leukaemia in young people was observed in rural communes with high population mixing (IRR=2.71; 95% CI: 1.10–6.35) (Table 3). The estimated IRR of leukaemia increased across categories of population mixing in rural ‘communes’ (*p* for trend=0.01). Similar results were obtained by conventional Poisson regression (1) for leukaemia among 0–24 year olds (Table 4

**Table 4:**
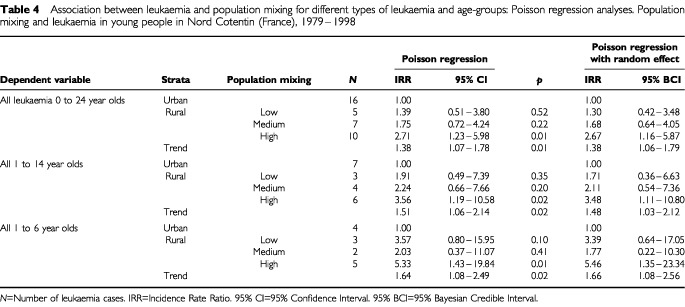
Association between leukaemia and population mixing for different types of leukaemia and age-groups: Poisson regression analyses. Population mixing and leukaemia in young people in Nord Cotentin (France), 1979–1998

). The effects of population mixing was more pronounced for ALL among children aged 1–14 years (IRR=3.56; 95% CI: 1.19–10.58 for the highest exposure category). It was even higher for ALL in the age group 1–6 years (IRR=5.33; 95% CI: 1.43–19.84).

The coefficients of the population mixing variable were somewhat smoothed by the introduction in the model of extra-Poisson variation (2) and the confidence intervals widened slightly (Table 4). The highest risk of leukaemia was still observed in the rural area most affected by population mixing either for all leukaemia in persons less than 25 years of age (IRR=2.67; 95% Bayesian credible interval, 95% BCI: 1.16–5.87), or for ALL among the 1–6 year olds (IRR=5.46; 95% BCI: 1.35–23.84).

## DISCUSSION

The Nord Cotentin region, where the La Hague plant is situated, has experienced during the years 1982 to 1992 one of the biggest building sites in Europe. For the years 1979 to 1998, we report a higher risk of leukaemia in young people in rural ‘communes’ the most affected by population mixing compared to urban ‘communes’. This risk was more pronounced for acute lymphoblastic leukaemia and for the 1–6 year old age group.

Our definition of the population mixing index is similar to the one used to investigate the association between childhood leukaemia and population movements brought about by the North Sea oil industry in Scotland (Kinlen *et al*, 1993). As the population mixing index of the Cumbrian study, it takes into account the in-coming of the workers from other parts of the country (Dickinson and Parker, 1999). This index reflects reasonably well the population movements for the La Hague construction site which took place from 1982 to 1992. Considering the facts that personal details were not available for Flamanville workers, that the two construction sites were planned in order to share the same construction workers and, that population mixing continued after 1992 for the regular running of the nuclear waste reprocessing plant, the same population mixing index was used as a surrogate for the entire period 1979–1998. Our population mixing index did not take into account population movements which could be due to migration of populations other than male construction workers, like other male workers involved in the construction of housing or education facilities, administrative or commercial people and relatives of the workers. The assumption was made that the intensity of the influx of male nuclear construction workers was proportional to the intensity of the general influx. Non differential misclassification bias could have occurred because of the missing values in the place of birth or in the address of the workers or the absence of registered address in the French department of ‘La Manche’ (5.5, 2.9 and 24.0% respectively). As linear inter-census projections in a context of an unstable population could have underestimated the expected numbers of leukaemia in the areas the most affected by population mixing (Cartwright *et al*, 1989), we developed a demographic method for the estimation of the person-years (Pottier, 1992).

Poisson regression with random effects allows a closer approximation to the geographical structure of the data. Random effects may be considered as surrogates for unknown or unmeasured area-level covariates (Best *et al*, 1999; Clayton *et al*, 1993; Mollié, 1996). If the unobserved risk factors exhibit spatial correlation, it may result in the non-independence of the true incidence rate ratio between contiguous areas. The clustering random effect is used here to allow for this spatial dependence. The heterogeneity random effect was used as a way to model unobserved risk factors which were not spatially correlated (Mollié, 1996). Unstructured heterogeneity had a greater influence on the IRR than spatial variation as assessed by the posterior mean of the heterogeneity and clustering components variance (data not shown) (Mollié, 1996). The three approaches to statistical modelling we used yielded very similar results.

As population mixing increased with proximity to the La Hague and Flamanville sites (Figure 2), the population mixing index could also be considered as a surrogate for the exposure to the radioactive discharges from the nuclear plants. In the Cumbrian study, the effects of proximity to the plant and population mixing have been dissociated by assessing the association between population movements and leukaemia in all parts of Cumbria but Seascale. The explicative model was then used to predict the number of cases attributable to population mixing in Seascale where the cluster of leukaemia was originally described (Dickinson and Parker, 1999). Our data set was too small to conduct a similar analysis and the arguments in favour of a causal effect of environmental radioactive discharges on the occurrence of leukaemia are weak (Rommens *et al*, 2000).

A higher risk of leukaemia in young people living in rural areas most affected by adult population mixing was observed in the study of the effect of the North sea oil industry in Scotland (Kinlen *et al*, 1993). The strength of this publication was the size of the area under study and the partition of the time period into pre-mixing, early post-mixing and later post-mixing periods. Because of the difficulty of estimating incidence rates of leukaemia before 1978 and the overlapping of the workers at the two main building sites, we were not able to split the study into several periods. In Cumbria as in Nord Cotentin, ALL in the younger age group is most affected by population movements although several differences between the studies should be noted: in Cumbria, the population movements variable is based on place of birth of parents and individual data have been aggregated at the electoral ward level to produce a collective measure of population mixing; analyses are based on the place of birth of the children and not the place of diagnosis; finally, person-years have been calculated with individual data (Dickinson and Parker, 1999).

Different population mixing variables have been used up to now: some are based on the influx of male workers or servicemen into an area (Kinlen and Balkwill, 2001; Kinlen *et al*, 1991, 1993, 1995), others are defined according to the proportion of parents born outside the area under study (Dickinson and Parker, 1999). Population density has also been used and is an alternative to population mixing variable (Alexander *et al*, 1999). All these variables may be thought of as surrogates for the exposure to an unknown risk factor, which makes difficult any causal interpretation. An infectious agent could be responsible for the association between population mixing and childhood leukaemia. Rural areas could be characterised by a lower level of natural immunisation to the infectious agent which may occur either because children have fewer opportunities for person-to-person transmission than in more urban areas, or because the population is not large enough to maintain the disease in an endemic form (Kinlen, 1988). Major population influx could be conducive to the spread of infectious agents and later, to the occurrence of childhood leukaemia. One intriguing observation is that, according to the Cumbrian study, among children born in the study area, those who were born to in-comers have a higher risk of developing leukaemia than those born to locally-born parents (Dickinson and Parker, 1999). This is unexpected if local populations are considered being more at risk (Kinlen, 1988), but may be indicative of the level of migration in a community. This could not be investigated in our data since details about the origin of the inhabitants were not available.

The population mixing hypothesis postulated a differential effect of population mixing in the urban and rural area according to the level of herd community (Kinlen, 1988). This is supported by a few studies with an effect of population mixing in the rural strata and no effect in the urban one although effect modification has never been statistically tested (Kinlen and Hudson, 1991; Kinlen *et al*, 1993, 1995). In our data, the effect of population mixing in urban areas was impossible to assess because only six ‘communes’ were classified as urban and because of their large size (average population of 15 300 inhabitants at the 1990 census). We noticed, in any events, a smaller risk of leukaemia in young people in urban areas and no population mixing effect when considering the whole data set without stratification on urban/rural status (data not shown). For urban areas, it might have been desirable to work at a smaller geographical unit than ‘communes’, but demographic and professional data were not available at this level.

The stronger association observed between population mixing and ALL among children aged 1–6 years than for all leukaemia among people aged 0–24 years could correspond either to a better definition of the health outcome supposing that population mixing may have a greater influence on ALL than on other leukaemia, or to a more accurate estimation of the exposure. In the latter case, infancy, if not pregnancy time, could be a critical exposure window for the effect of population mixing on the occurrence of leukaemia. This is in accordance with the two-stage model derived from experimental data on cellular transformation and leukemogenesis: a minimum of two independent and sequential mutations are likely to be necessary to produce acute leukaemia: the leukaemia could be initiated *in utero* but may require a post natal event for full expression as acute leukaemia (Greaves and Alexander, 1993).
